# Neuromyelitis optica study model based on chronic infusion of autoantibodies in rat cerebrospinal fluid

**DOI:** 10.1186/s12974-016-0577-8

**Published:** 2016-05-18

**Authors:** R. Marignier, A. Ruiz, S. Cavagna, A. Nicole, C. Watrin, M. Touret, S. Parrot, G. Malleret, C. Peyron, C. Benetollo, N. Auvergnon, S. Vukusic, P. Giraudon

**Affiliations:** INSERM U1028, CNRS UMR 5292, Center for Research in Neuroscience of Lyon, Lyon, France; Université Lyon 1, Université de Lyon, Lyon, France; Service de Neurologie A, Eugène Devic EDMUS Foundation Against Multiple Sclerosis, Observatoire Français de la Sclérose en Plaques, Hôpital Neurologique Pierre Wertheimer, Hospices Civils de Lyon, 59 Boulevard Pinel, 69677 Lyon-Bron cedex, France

**Keywords:** Neuromyelitis optica, Aquaporin-4, Astrocyte, Glutamate, Animal model, Autoantibodies, Autoimmune diseases

## Abstract

**Background:**

Devic’s neuromyelitis optica (NMO) is an autoimmune astrocytopathy, associated with central nervous system inflammation, demyelination, and neuronal injury. Several studies confirmed that autoantibodies directed against aquaporin-4 (AQP4-IgG) are relevant in the pathogenesis of NMO, mainly through complement-dependent toxicity leading to astrocyte death. However, the effect of the autoantibody per se and the exact role of intrathecal AQP4-IgG are still controversial.

**Methods:**

To explore the intrinsic effect of intrathecal AQP4-IgG, independent from additional inflammatory effector mechanisms, and to evaluate its clinical impact, we developed a new animal model, based on a prolonged infusion of purified immunoglobulins from NMO patient (IgG^AQP4+^, NMO-rat) and healthy individual as control (Control-rat) in the cerebrospinal fluid (CSF) of live rats.

**Results:**

We showed that CSF infusion of purified immunoglobulins led to diffusion in the brain, spinal cord, and optic nerves, the targeted structures in NMO. This was associated with astrocyte alteration in NMO-rats characterized by loss of aquaporin-4 expression in the spinal cord and the optic nerves compared to the Control-rats (*p* = 0.001 and *p* = 0.02, respectively).

In addition, glutamate uptake tested on vigil rats was dramatically reduced in NMO-rats (*p* = 0.001) suggesting that astrocytopathy occurred in response to AQP4-IgG diffusion. In parallel, myelin was altered, as shown by the decrease of myelin basic protein staining by up to 46 and 22 % in the gray and white matter of the NMO-rats spinal cord, respectively (*p* = 0.03). Loss of neurofilament positive axons in NMO-rats (*p* = 0.003) revealed alteration of axonal integrity. Then, we investigated the clinical consequences of such alterations on the motor behavior of the NMO-rats. In a rotarod test, NMO-rats performance was lower compared to the controls (*p* = 0.0182). AQP4 expression, and myelin and axonal integrity were preserved in AQP4-IgG-depleted condition. We did not find a major immune cell infiltration and microglial activation nor complement deposition in the central nervous system, in our model.

**Conclusions:**

We establish a link between motor-deficit, NMO-like lesions and astrocytopathy mediated by intrathecal AQP4-IgG. Our study validates the concept of the intrinsic effect of autoantibody against surface antigens and offers a model for testing antibody and astrocyte-targeted therapies in NMO.

**Electronic supplementary material:**

The online version of this article (doi:10.1186/s12974-016-0577-8) contains supplementary material, which is available to authorized users.

## Background

Devic’s neuromyelitis optica (NMO) is now considered a primary astrocyte disease associated with central nervous system (CNS) inflammation, demyelination, and neuronal injury [1–5]. Aquaporin-4 autoantibody (AQP4-IgG) plays a major role in this astrocytopathy. Several clinical, immunological, and pathological studies have demonstrated that AQP4-IgG is pathogenic through complement-dependent astrocyte toxicity [6–9].

However, autoantibodies against membrane antigen can also act directly as receptor agonists/antagonists or modulate antigen density and trigger cell dysfunction, prior or in addition to complement activation or secondary inflammatory cell-mediated response [10]. These mechanisms probably take part, to some extent, in NMO. In fact, some patients experience fully reversible clinical symptoms and MRI abnormalities, suggesting a transient modulatory phenomenon rather than a destructive one [11]. Modulation of AQP4 membrane expression has also been demonstrated in several study models and associated with increase blood-brain barrier permeability, water balance disruption, and glutamate excitotoxicity [12–14]. Recent detailed study of NMO pathology showed different lesion types, some associated to complement and immune cells infiltration but others with selective astrocyte loss in the absence of complement activation and granulocyte infiltration [15].

These data strongly support the idea of an intrinsic modulatory effect of AQP4-IgG in NMO. Studies providing evidence for a direct pathogenic role of AQP4-IgG were mainly performed in co-existing inflammatory conditions [16–20]. Recently, Geis et al. investigated the effect of the autoantibody itself by performing repeated local injection of high dose of purified immunoglobulins from AQP4-IgG-positive patients in the spinal cord of rats. They found a reversible downregulation of AQP4 with preserved astrocyte, axon, and myelin in contact to inflammatory reaction at the site of injection, associated with transient clinical changes and correlated it with “penumbra-like” lesions recently reported in NMO [21].

To explore more widely the intrinsic immunopathogenic effect of AQP4-IgG and its functional and morphological consequence not only on astrocyte but also on myelin and axons and to evaluate its clinical impact, we have developed a new animal model of NMO based on a chronic infusion of “physiological dose” of purified immunoglobulins (IgG) from NMO patient and healthy individual, into the cerebrospinal fluid (CSF) of live rats.

Here, we show that chronic in vivo CSF infusion of purified AQP4-IgG alone leads to diffusion of all CNS structures and induces NMO-like lesions mainly in the optic nerve and the spinal cord. These histopathological changes are associated with motor behavioral changes, probably via astrocytopathy and glutamate homeostasis disturbance, and happen in the absence of the major complement deposition or immune cell infiltration.

## Methods

### Patients, IgG purification of samples for animal experiments

Sera and/or plasmapheresis were obtained from three patients with NMO spectrum disorder [22] selected from the French NMO cohort NOMADMUS and stored at NeuroBioTec (Biological Resource Centre of the *Hospices Civils de Lyon*). All sera were tested positive for AQP4-IgG [23]. We also used control sera collected from three healthy blood donors at *Etablissement Français du Sang*. IgG were purified from the sera and plasmapheresis on Protein-A Sepharose 4 Fast Flow^TM^ beads (P9424 Sigma-Aldrich ®), eluted with glycine 0.1 M buffer pH 2.8 and then neutralized in Tris 1.5 M pH 8.8. The samples were concentrated to obtain an IgG concentration of 1.5–2 mg/mL. We termed IgG^AQP4+^1–3 as the total IgG isolated from the three AQP4-IgG-seropositive sera and IgG^Control^1–3 as the total IgG isolated from the three sera of the healthy donors. IgG^AQP4+^1–3 displayed a strong positive anti-AQP4 binding pattern on HEK-AQP4 cells using flow cytometry. Prior to their use for brain infusion, purified IgG were tested for immunoreactivity on rat CNS tissue. They stained astrocytes and co-localized it with glial fibrillary acidic protein (GFAP) and AQP4 in the rat brain, spinal cord, and optic nerve (Additional file 1: Figure S1A). IgG^Control^ showed no specific staining on astrocyte (not shown). The pathogenicity of IgG^AQP4+^ was tested on cultured astrocytes. As shown by Western blot analysis, IgG^AQP4+^ decreased AQP4 expression in the total astrocyte lysate (Additional file 1: Figure S1B) (51 ± 21 decrease versus 1.5 ± 3 % decrease with IgG^Control^, *p* = 0.008) and membrane preparation (Additional file 1: Figure S1C) following 24-h exposure.

### AQP4-IgG depletion

Human embryonic kidney-293 cells stably transfected with human AQP4-M23 plasmid (HEK-AQP4 cells engineered by CreaCell®, Grenoble France) were seeded in six-well plate at a density of 1 × 0^6^ cells. IgG^AQP4+^ was added in each well to live HEK-AQP4 cells for 20 min per well at 37 °C, CO_2_ atmosphere and gentle rotation. Contact with cells was performed six times. After cell contact, depleted IgG^AQP4+^ was removed, centrifuged for 15 min at 12.000 rpm, then tested for AQP4 antibody titer by flow cytometry, as already described [24]. Adsorption of IgG^AQP4+^ on HEK-AQP4 cells decreased AQP4 antibody level by almost 40 % (Fig. 8e). Adsorbed IgG^AQP4+^ were termed IgG^AQP4+dep^.

### Animals, surgery and intraventricular infusion

For the purpose of this work, a total of 65 Oncins France Strain A (OFA) rats were included. Animal experiments were carried out in strict accordance with the European Directive 2010/63/UE and follows the Animal Research: Reporting of In Vivo Experiments (ARRIVE) guidelines on the protection of animals used for scientific purposes. Adult male OFA rats at 280 g (Janvier; Le Genest Saint Isle, France) were acclimated to the new environment for 7 days prior to the start of the experiment. The local Lyon 1 University Animal Care Committee has approved all the experimental procedures on animals (BH2012-80 project). A total of 33 rats received IgG^AQP4+^ (termed NMO-rats), 15 rats received IgG^Control^ (termed Control-rats), 15 rats received NaCl (termed Saline-rats). One experimental group was dedicated to immunodetection analyses (immunohistochemistry (IHC), Western blot) (eight NMO-rats, eight Control-rats, one Saline-rat). Two rats of each IgG group were sacrificed on day 14 (D14) following the beginning of perfusion, the others being sacrificed on D7. The second experimental group was dedicated to in vivo analysis of glutamate uptake, performed on D7 (four NMO-rats; four Control-rats). The third experimental group was dedicated to behavioral study performed on D3, D7, D10, D14, and D21 (ten Saline-rats, ten NMO-rats). A fourth group was dedicated to transmission electron microscopy analyses (five NMO-rats, one Control-rat, two Saline-rats). In addition, several control experiments included (i) infusion of IgG following AQP4-IgG depletion (three NMO-rat^AQP4+dep^); (ii) 2-day treatment with cobra venom (two rats); (iii) being together with five NMO-rats. All were examined using IHC.

For surgery, the rats were continuously anesthetized with volatile isoflurane (Aerrane, Baxter SAS, France) and mounted conventionally in a stereotaxic frame (David Kopf, Epinay-sur-Seine, France). The scalp was shaved and incised, and the skull was cleaned and dried. Then, 400 μg of purified IgG (IgG^AQP4+^, IgG^Control^, IgG^AQP4+dep^; 200 μL at 2 μg/μL) or 200 μL NaCl were infused into the CSF during 7 days at a rate of 1 μL/h, using a sterile mini-osmotic pump implanted subcutaneously between the rat scapulae and previously connected to a brain infusion kit (osmotic pump ALZET, Charles Rivers, France). The cannula of the brain infusion kit was lowered into the right lateral ventricle using stereotaxic coordinates (anteroposterior (AP) −0.7 mm; mediolateral (ML) −1.7 mm from Bregma; depth, 5 mm from the skull surface) and strongly fixed to the skull with superglue. After surgery, the rats were maintained on a 12-h light/dark cycle and housed in transparent individual cages with food and water ad libitum for up to 21 days, depending on the conducted experiments.

### Tissue preparation and harvesting, immunohistochemistry, and immunoblot

At the end of brain infusion (D7) and on D14 and D21, the rats were anesthetized and received intracardiac perfusion with 100 mL phosphate-buffered saline (PBS; 0.1 M pH 7.4) to remove blood cells. The rats were then killed with a lethal dose of pentobarbital. Following removal from the skull and laminectomy, the brain, spinal cord, and the optic nerves were frozen in isopentane at −30 °C and stored at −80 °C. Just before IHC, neural tissue was embedded in Tissue-Tek OCT® compound for optimal cutting and prepared as 8–10-μm sections on a cryomicrotome. These slices were used for immunodetection. Contact with primary antibody (4 °C, overnight) was followed by three washes and 5-h contact at room temperature with Alexa-488-, Alexa-555-, or Alexa-647-labeled anti-mouse, anti-rabbit, and anti-human IgG antibody (Molecular Probes A11013, USA).

Following IHC, the tissue sections were viewed and digitized with an Axio Imager Z1 apotome technology (Zeiss, Oberkochen, Germany) by an examiner blinded to treatment allocation with the appropriate filter settings using AxioVision Rel 4.8 software. Analyses were performed with ImageJ 1.4 software (Wayne Rasband NIH, USA, public domain). For IHC signal intensity analyses, all immunostaining were digitized with the identical microscope and software settings. For quantification, a standardized area was analyzed in different CNS structures of interest: the spinal cord gray and white matter, the optic nerve, the corpus callosum, and the periventricular areas. Means of intensity values of each animal were then taken for further analyses. For axon analyses, axons in the immunostained spinal cord slices (neurofilament (NF)-M detection) were digitized, organized according their surface (10 to 140 μm^2^) and counted using ImageJ software.

For immunoblot (Western blot), tissue punches from the periventricular areas, corpus callosum, and spinal cord were dissociated using ultrasound fragmentation in homogenization buffer containing Tris 20 mM, EDTA 1 mM, EGTA 1 mM, sucrose 10 % pH 7.4, protease inhibitor (Complete EDTA free, Roche Diagnostics GmbH 69298 Mannheim, Germany) and phosphatases inhibitors (sodium fluoride 10 mM, sodium pyrophosphate 2 mM, sodium beta-glycerophosphate 2 mM, sodium orthovanadate 2 mM). The samples were screened for protein content (Qubit® Protein Assay Kits Q33212 Molecular Probes®) then examined for protein expression using Western blotting as described [25]. Briefly, proteins were separated on SDS-PAGE gels (1–5 μg per lane, 10 % Criterion™ XT Bis-Tris Gel and 4–12 % Criterion™ XT Bis-Tris Gel, © Bio-Rad Laboratories Inc Hercules CA 94547), transferred on nitrocellulose (Protran® Nitrocellulose Hybridization Transfer Membrane 0.45 μm Whatman®), immunodetected (antibody diluted in Tris buffer saline (TBS) containing 1 % non-fat dry milk and 0.1 % Tween-20) using primary antibody (4 °C overnight) and anti-IgG antibody (1-h room temperature), and revealed using chemiluminescence (Immobilon Western Chemiluminescent HRP Substrate WBKLS0500 Millipore Merck KGaA Darmstadt Germany; Amersham Hyperfilm™ ECL GE Healthcare UK Ltd Buckinghamshire UK). Band density was evaluated using ImageJ software.

### Transmission electron microscopy

Transmission electron microscopy (TEM) was performed at the CIQLE facilities—*Centre d'Imagerie Quantitative Lyon-Est* (http://sfrsantelyonest.univ-lyon1.fr/plateau31-ciqle.html). The rats were perfused with a washing solution containing cacodylate and CaCl_2_, then with a fixing solution 2 % paraformaldehyde, 2 % glutaraldehyde, cacodylate, and CaCl_2_. The optic nerve and fragment of 1 mm^3^ of the spinal cord were postfixed in the fixation solution overnight at 4 °C, then dehydrated in alcohol containing 0.1 % tannic acid and embedded in Epon resin. Ultrathin sections (70-nm thick) were cut with a Reichert Ultracut E (Leica) ultra-microtome, mounted on 200 mesh copper grids coated with 1:1000 poly-lysine and contrasted with uranyl acetate and lead citrate. The sections were observed in a Jeol 1400JEM (Tokyo, Japan) transmission electron microscope operating at 80 kV equipped with an Orius 600 camera and Digital Micrograph. Tissue examination was performed on five NMO-rats, one Control-rat, and two Saline-rats. In each treatment condition, ≈20 areas in the optic nerve and spinal cord were examined.

### Astrocyte primary culture and immunocytochemistry

Primary glial cultures were obtained by mechanical disruption of microdissected cortices from 1-day-old rat pups, as previously described [24]. Dissociated cells were diluted to a density of 2.10^5^ cells/mL in Dulbecco’s modified Eagle medium (DMEM) minimal essential Glutamax medium (Gibco, Life Technologies, France) containing 4.5 g/L glucose, supplemented with 20 % heat-inactivated fetal calf serum and gentamycin (1 μg/mL). The cells were seeded in six-well plates and LabTek slides pre-coated with poly-L-lysine (3 μg/mL in 0.1 M borate buffer, pH 8.4) and incubated at 37 °C in a moist 5 % CO_2_, 95 % air atmosphere. The medium was changed every 3 days after plating (10 % fetal calf serum) and treated with cytosine arabinoside (AraC, 25 nM, Sigma-Aldrich) to eliminate microglia and oligodendrocyte and to obtain pure cultured astrocytes. Immunocytochemistry was performed on acetone-fixed cells (10 min, −20 °C) or paraformaldehyde (4 %, 15 min) (primary antibody 45 min 37 °C, fluorochrome-labeled anti-IgG antibody 30 min room temperature). The culture was treated with IgG^AQP4+^ and IgG^Control^ (75 μg/mL) for 24 h before astrocyte protein analysis. Subcellular fractioning of astrocyte was performed using the ProteoExtract® subcellular proteome extraction kit (Merck Millipore) and proteins analyzed using Western blot.

### Immunodetection

The following antibodies were used for immunodetection on the tissue samples and cell culture and lysates. The primary antibodies to AQP4 are as follows: rabbit AB2218, Merck© KGaA, Darmstadt, Germany; beta-actin, MoAb A1978, Sigma-Aldrich®, St. Louis, MO 63103, USA; GFAP: rabbit Z0334, Dako Denmark A/S; GLT1-EAAT2: Mouse anti-EAAT2, ab77039, Abcam®; Iba-1: rabbit 019-19741, Wako Chemicals USA, Inc; MBP: MoAb MCA70, AbDSerotec©, Bio-Rad Laboratories, Inc; Neurofilament Heavy (NF-H): rabbit AHP2259GA, AbDSerotec© Bio-Rad Laboratories, Inc; Neurofilament Medium (NF-M): MoAb ab7794; phospho-Neurofilament: SMI34, MoAb ab24571 Abcam® Cambridge, UK; human IgG: Biotin-SP-conjugated affinityPure donkey anti-human IgG 709-065-149 Jackson ImmunoResearch; CD45 pan lymphocyte: mouse clone Ox-1, BD Pharmingen™; CD45RA: mouse clone OX33, BD Pharmingen™; CD8: mouse antibody 554854 BD Pharmingen; and olig2: rabbit antibody AB9610, Millipore. The secondary antibodies are: Peroxidase-AffiniPure F(ab’)2 fragment donkey anti-mouse IgG(H+L) 715-036-151; Peroxidase-affiniPure F(ab’)2 fragment donkey anti-rabbit IgG(H+L) 711-036-152 Jackson ImmunoResearch Laboratories, Inc. west Grove PA; Alexa Fluor®488 goat anti-mouse IgG A11029; Alexa Fluor®555 goat anti-mouse IgG A21424; Alexa Fluor®488 goat anti-rabbit IgG A11034 ; and Alexa Fluor®455 goat anti-rabbit IgG A21429 from Molecular Probes Inc.

### In vivo microdialysis and measurement of glutamate uptake

A method previously described by our group [26] was used to assess in vivo glutamate uptake in the rat hippocampus. Concentric microdialysis probes were thus constructed and, before implantation, were perfused at a rate of 1 μL/min with artificial cerebrospinal fluid (aCSF) (149 mM NaCl, 2.80 mM KCl, 1.2 mM MgCl_2_, 1.2 mM CaCl_2_, 2.78 mM phosphate buffer, pH 7.4). A probe was implanted into the guide cannula of a freely moving rat placed in a plexiglass cylinder, the inlet and outlet of the probe being connected to a liquid swivel (Instech Solomon, USA). The rate of infusion was 2 μL/min. For each dialysis experiments, at least 3 h was allowed to elapse after microdialysis probe implantation before collecting basal samples. At the end of the experiment, the rats were killed with a lethal dose of pentobarbital, and the placement of the cannula was verified on the removed brain [27].

Uptake experiments were performed by switching the microdialysis probe perfusion fluid for 10 min to aCSF containing 250 μM ^3^H-glutamate (GLU) (15 Ci/mMol) and 120 μM ^14^C-mannitol (MAN) (60 mCi/mMol) (PerkinElmer Life and Analytical Sciences, USA), samples of microdialysis probe effluent being collected every 2 min for dual-label scintillation counting. The relative GLU uptake was determined using the “recovery” versus “time” curves for ^3^H-GLU and ^14^C-MAN used as the reference, as cells do not take it up. The recovery is the counts exiting the probe/input counts ratio (Eqs. 1 and 2). The cellular extraction fraction for Glu corrected for mannitol is calculated as in Eq. 3.

1 $$ {\mathrm{R}}_{\mathrm{MAN}}=\mathrm{MAN}\ \mathrm{outlet}/\mathrm{MAN}\ \mathrm{inlet} $$

2 $$ {\mathrm{R}}_{\mathrm{GLU}}=\mathrm{G}\mathrm{L}\mathrm{U}\ \mathrm{outlet}/\mathrm{G}\mathrm{L}\mathrm{U}\ \mathrm{inlet} $$

3 $$ {\mathrm{E}}_{\mathrm{L}\hbox{-} \mathrm{G}\mathrm{L}\mathrm{U}}={\mathrm{R}}_{\mathrm{MAN}}-{\mathrm{R}}_{\mathrm{GLU}}/{\mathrm{R}}_{\mathrm{MAN}} $$

### Motor behavior analyses

The accelerating rotarod apparatus [28] was used to measure motor coordination. Prior to surgery, the animals were pre-trained for three consecutive days, receiving two trials per day with a 4-h inter-trial interval until they reached the criteria 2-min stay on the rotating rod. The animals not reaching the criteria were excluded from the behavioral experiments. There was no weight loss for rats along the experimentation (Additional file 2: Figure S2A). The NMO-rats (*n* = 10) and Saline-rats (*n* = 10) were thus pre-trained to balance on the accelerating rod prior to surgery and then tested at D3, D7, D10, D14, D17, and D21 postsurgery. The latency to fall during the observation period was recorded and normalized to D0 (prior to surgery) performance. Prior to the rotarod test, the rats were examined for their ability to grip a suspension bar. There was no clear difference between the two groups. An observer blinded to the group assignment scored the amount of time the animal spent on the rotarod. In order to assess the natural walking pattern of each animal, motor behavior was also analyzed in a non-forced complete stepping sequence task on D0, D7, D14, and D21. A walking apparatus was thus adapted from Metz and Whishaw [29]. In this task, the rats were invited to walk in a corridor to mark their footprints. This corridor consisted of a flat floor (1.0 mL × 9.5 cm l × 19.0 cm) closed by two wooded sidewalls. At one end of the corridor, the rat could be placed in a resting box where an ink stamp placed on the floor allowed the labeling of the rat’s fingers and paws. The marks left by the rat’s footprints as it moved along the corridor were collected on paper. A manual scoring was determined for the analysis of footprints. Distance between anterior legs, distance between posterior legs, width between anterior legs, width between posterior legs, and difference in finger gauge of anterior legs were thus evaluated (Additional file 3: Figure S3B). Using these footprints, the experimenter was thus allowed to compute the trajectory of the rat and to assess if this trajectory deviated from the linear trajectory initially taken by the rat when entering the corridor at D0, D7, D14, and D21. Figure 7b shows the percentage of shift from D0 deviation. Using this analysis of the left forepaw prints, deviation from the line trajectory taken by the rat once it entered the corridor was computed and scored on D0, D7, D14, and D21, and results were expressed as a percentage of shifts to D0 performance (deviation observed prior to surgery). An observer masked to group assignment analyzed the natural walking of each animal.

### Statistical analyses

Investigators blinded for treatment condition performed analyses of immunodetection, Western blot and electron microscopy. Following quantification of fluorescence intensity (immunodetection) and Western blot signal, data are presented as mean ± SD. Considering the non-normal distribution, we used the non-parametric Mann-Whitney test to compare two groups and one-way ANOVA to compare more than two groups. The levels of significance were indicated as follow: *p < 0.05, **p < 0.01, and *** *p* < 0.001. For glutamate uptake measurement, we compared differences in uptake between the mean ± SEM for the NMO-rats (*n* = 4) and the Control-rats (*n* = 4) using the unpaired Student’s *t* test and a level of significance of *p* < 0.05. Computation was performed using Prism 5.0 GraphPad.Inc software. For behavioral analysis, we used repeated measures ANOVAs to assess the variation of performance over days.

## Results

### IgG^AQP4+^ infused in the rat CSF diffused in the CNS and induced astrocytopathy

**Fig. 1:**
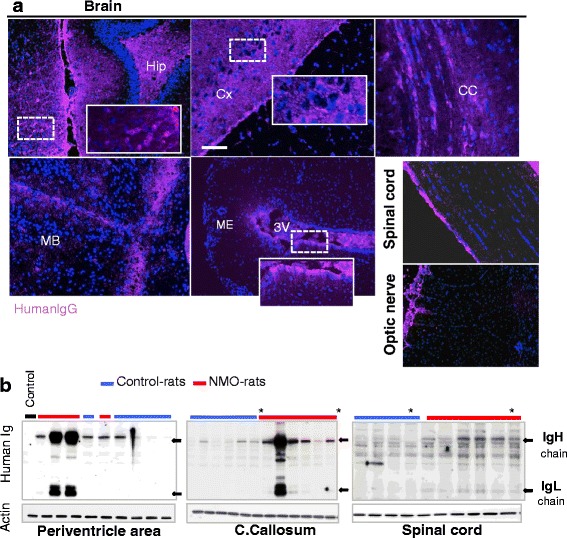
Human IgG chronically infused in the rat brain ventricle widely diffused in the neural tissues. **a** Detection of human IgG in the rat brain, spinal cord, and optic nerve using immunohistochemistry (rat infused with IgG^AQP4+^2, D7 as and example). *Scale bar* 10 μm. *Hip* hippocampus, *Cx* cortex, *CC* corpus callosum, *MB* midbrain, *ME* median eminence, *3V* third ventricle. **b** Detection of human immunoglobulin IgH and IgL chains (*black arrows*) accumulated in neural tissues of NMO-rats (received IgG-NMO) and Control-rats (received control IgG) ((*asterisk*) D7 and D14) using Western blot (actin as protein deposition control, same blot. Control: neural tissue from rat receiving saline buffer)

To validate our model of chronic infusion of autoantibody in rat CSF, we controlled the presence of human IgG in neural tissues of eight rats infused with IgG^AQP4+^ (NMO-rats) and eight rats infused with IgG^Control^ (Control-rats) on D7 and D14. Human IgG was detected by IHC in different brain structures and in the optic nerve and spinal cord of the NMO-rats (Fig. 1a) and the Control-rats. Western blot performed on neural tissue from the NMO-rats and the Control-rats showed accumulation, at various levels, of human IgG (H and L chains) in the infused CNS (Fig. 1b). Altogether, we detected the diffusion of CSF-infused human IgG in the rat CNS.

**Fig. 2:**
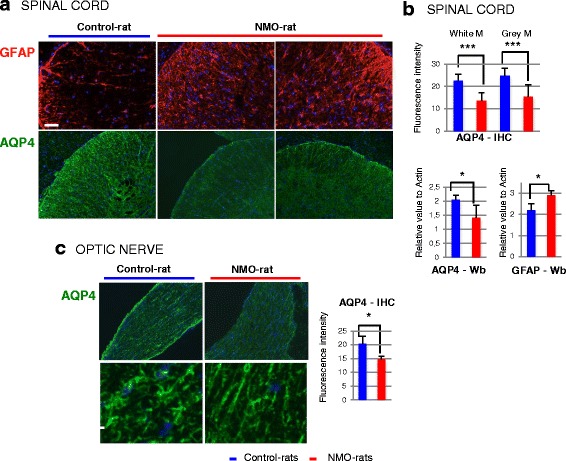
Chronic infusion of IgG^AQP4+^ decreased AQP4 expression in the rat spinal cord and optic nerve. **a** Example of AQP4 loss with preserved GFAP-positive astrocytes in the spinal cord of the NMO-rat compared to the Control-rat (D7, rat infused with IgG^AQP4+^1,2 and IgG^Control^1,2 as an example) *IHC* immunohistochemistry. *Scale bars* = 20 μm. **b** Confirmation of AQP4 loss using evaluation of AQP4 expression in the white and gray matter of the NMO-rat spinal cord (*n* = 5) compared to the Control-rat (*n* = 4), by IHC (fluorescence intensity) and Western blot (Wb, relative to actin). In parallel, GFAP expression is enhanced (Wb). **c** Loss of AQP4 expression in the optic nerve of the NMO-rat as shown by IHC and evaluation of fluorescence intensity (*p < 0.05; ***p < 0.001, ANOVA)

We further searched for astrocyte alteration, focusing our analysis on AQP4 expression in the spinal cord and optic nerve of the NMO-rats (*n* = 8) and the Control-rats (*n* = 8) examined at D7 and D14. IHC showed a decreased AQP4 expression in the spinal cord of the NMO-rats compared to the Control-rats (Fig. 2a), a decrease confirmed by evaluation of the AQP4 level (fluorescent intensity), in the white and gray matter (Fig. 2b) (*p* = 0.001). Western blot (Wb) performed on all rats (Fig. 2b) confirmed the AQP4 decrease in the NMO-rats (*p* = 0.02). AQP4 decrease was not due to astrocyte loss as IHC and Western blot rather detected an increased GFAP expression (Fig. 2a, b (*p* = 0.02), respectively), suggestive of reactive cells. In the optic nerve, IHC performed and quantified on all rats, detected AQP4 loss in the NMO-rats compared to the Control-rats (Fig. 2c, *p* = 0.02). Globally, AQP4 loss in the NMO-rats was not restricted to perivascular area and occurred without marked astrocyte loss. Decrease of AQP4 expression also occurred in other myelinated CNS structures of the NMO-rats, notably *tractus optica* and *corpus callosum* (not shown).

**Fig. 3:**
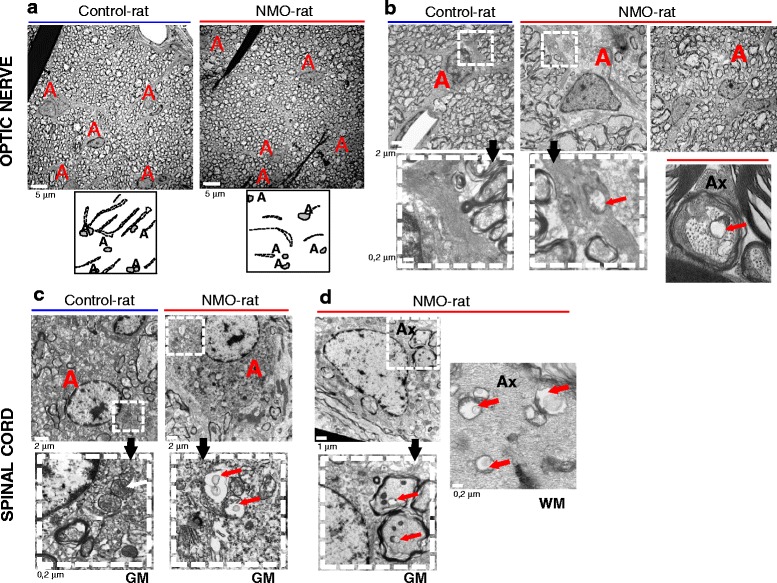
Astrocyte and axonal alteration detected at the ultrastructural level in the optic nerve and spinal cord of NMO-rats. **a**, **b** Transmission electron microscopy on the rat optic nerve (D7, rat infused with IgG^AQP4+^3 as an example). **a** Loss of astrocytic processes in the NMO-rat compared to the Control-rat, as shown in the corresponding drawings of astrocytic processes (*dotted line*). *A* astrocyte nucleus. **b** Axon bundle disorganization and altered mitochondria in astrocytic processes in NMO-rat (*red arrow*). Altered mitochondria were also present in axon (*Ax*). **c**, **d** Transmission electron microscopy on the rat spinal cord, in the gray (*GM*) and white matter (WM): altered mitochondria in NMO-rat (*red arrows*) compared to the Control-rat **c** in astrocytes and **d** in axon (*Ax*)

**Fig. 4:**
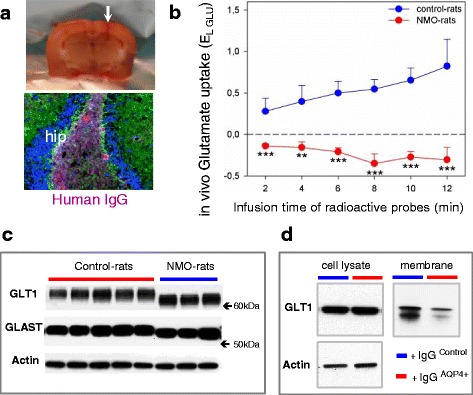
Functional astrocyte alteration in NMO-rat. **a** Rats were implanted (*white arrow*) with a microdialysis probe in the hippocampus (*hip*), where infused human IgG accumulated (*lower figure*) (D7). **b** Decreased glutamate uptake in NMO-rats detected using i*n vivo* uptake of exogenous radioactive glutamate (^3^H-GLU) and mannitol (^14^C-MAN, used as the reference molecule not taken up by cells) in the brain of the vigil NMO-rats (IgG^AQP4+^2, *n* = 4) and the Control-rats (IgG^Control^2, *n* = 4) (10-min infusion and collection of microdialysis probe effluent every 2 min for dual-label counting). The relative glutamate uptake was determined by Eq. 3 (E_L-GLU_ = R_MAN_ − R_GLU_/R_MAN_) using the “recapture” curves for ^3^H-GLU and ^14^C-MAN and expressed as the mean ± SEM. **c** Change in the apparent molecular weight of the glutamate transporters GLT1 and GLAST detected by Western blot in the NMO-rats compared to the Control-rats (periventricular area, actin as control of protein deposition). **d** Cell fractioning of cultured astrocytes treated with IgG^AQP4+^ (24 h): loss of GLT1 expression in the membrane fraction although the total cell lysate remained unchanged (Western blot). No similar effect with IgG^Control^ treatment

Astrocyte integrity was examined at the ultrastructural level (transmission electron microscopy) in the spinal cord and optic nerve at D7 (Fig. 3). In the optic nerve, the more striking modification is the loss of astrocytic processes delimiting axons bundles in the NMO-rats compared to the Control-rats (Fig. 3a, b). The presence of damaged mitochondria in the cytoplasm and processes of astrocytes together with tissue disorganization complemented this observation and suggested cell dysfunction (Fig. 3b). Damaged mitochondria were also observed in the axons of the optic nerve (Fig. 3b). In the spinal cord (Fig. 3c, d), damaged mitochondria are present in some astrocytes and axons within the gray matter (GM) and white matter (WM). Functional integrity of astrocyte was then tested by microdialysis on vigil IgG-infused rats, a method that reveal the ability of astrocytes to uptake glutamate. The microdialysis probe was implanted in the hippocampus, a brain structure rich in glutamate and where infused human IgG accumulated (Fig. 4a). A mixed solution of ^3^H-glutamate and ^14^C-mannitol (mannitol used as a non-uptaken molecular control) was infused in NMO-rats (*n* = 4) and Control-rats (*n* = 4). At D7, analysis of the samples collected in the rat brain every 2 min showed that glutamate uptake was dramatically reduced in the NMO-rats compared to the Control-rats (*p* = 0.001) (Fig. 4b). To understand the basis of such a functional alteration, we analyzed the expression of the glutamate transporters glutamate transporter type 1 (GLT1) and glutamate aspartate transporter (GLAST) in the implanted brain area, using Western blot. Compared to the Control-rats, a shift in the molecular weight (≈3 kDa) of GLT1 and GLAST was detected in the NMO-rats, suggesting protein modification (Fig. 4c). Analyzing subcellular distribution of GLT1 in cultured astrocytes after 24-h contact with IgG^AQP4+^, we observed that GLT1 level was not modified in the total cell lysate but was deeply reduced in the membrane cell fraction (Fig. 4d). Treatment with IgG^Control^ did not modify GLT1 expression. Thus, protein modification and decreased membrane expression of glutamate transporters induced by treatment with IgG^AQP4+^ could support the decreased glutamate uptake in the NMO-rats.

### IgG^AQP4+^-induced astrocytopathy is associated with myelin alteration, axonal damage, and motor impairment

**Fig. 5:**
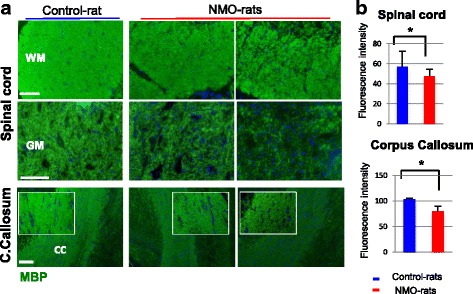
Myelin alteration in the spinal cord and corpus callosum of NMO-rat. **a** Myelin alteration detected by myelin basic protein (MBP) immunostaining in the spinal cord (gray (*GM*) and white matter (WM)) and corpus callosum (*CC*) of the NMO-rats compared to the Control-rat (D7, rats infused with IgG^AQP4+^1,2 and IgG^Control^2 as example). **b** MBP decreased expression in the spinal cord and corpus callosum of the NMO-rats (*n* = 4) compared to the Control-rats (*n* = 5), as shown by evaluation of fluorescence intensity (*p* < 0.05, ANOVA). *Scale bars* = 20 μm

Dysfunction of astrocyte induced by AQP4-IgG could have a serious consequence on myelin and axon. We first investigated myelin integrity in the spinal cord and optic nerve of the IgG-infused rats (D7, D14). Immunodetection of myelin basic protein (MBP) showed a diffuse myelin alteration in the GM and WM of the spinal cord of the NMO-rats examined at D7 (Fig. 5a). Myelin alteration also occurred in the corpus callosum of the NMO-rats (Fig. 5a) but was not obvious in the optic nerve (not shown). Evaluation of MBP staining intensity in the rat spinal cord (Fig. 5b, *p* = 0.03) confirmed its decrease in the NMO-rats (*n* = 4) compared to the Control-rats (*n* = 5), with a marked alteration in the GM (mean decrease, 30 ± 9.9 % in the GM versus 17.4 ± 7 % in the WM). Myelin alteration was confirmed at D21 by proteolipid protein (PLP) labeling (mean decrease of fluorescence intensity, 39.5 ± 0.1 %) and associated the loss of olig2-positive oligodendrocytes in the NMO-rats compared to the Control-rats (39.6 ± 7.6 versus 66.8 ± 16 positive cells per field, respectively, *p* < 0000,1). Oligodendrocyte loss was not detected at D7.

**Fig. 6:**
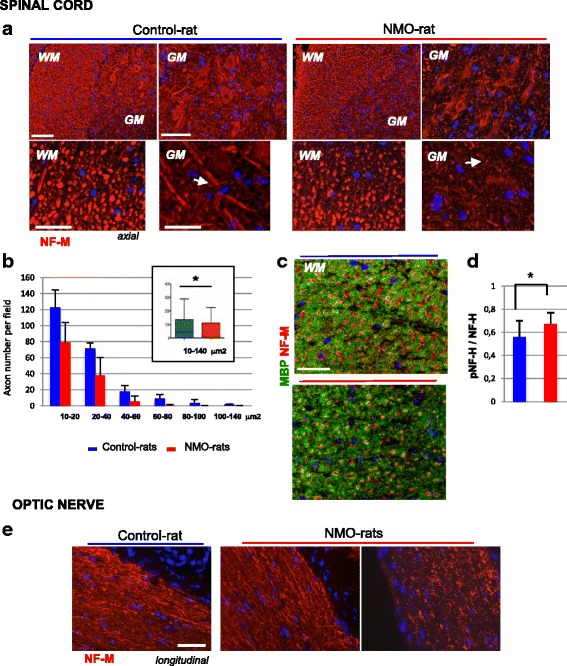
Axonal damage and loss in the spinal cord and optic nerve of the NMO-rat. **a** Axon injury detected in the NMO-rat compared to the Control-rat (rats infused with IgG^AQP4+^2 and IgG^Control^2, D7) using neurofilament immunostaining: reduced number of axons detected as NF-M-positive spots in the white matter (WM); fragmentation and reduced axon thickness in the gray matter (GM). **b** Classification (10–20 to 100–140 μm^2^, ImageJ) and quantification (mean by field) of NF-M-positive spots in the spinal cord of the NMO-rats (*n* = 6) compared to the Control-rats (*n* = 6): loss of axons with 60–140 μm^2^ sections in the NMO-rats (in cart: evaluation of the total axon number, *p* = 0.03). **c** Co-detection of myelin alteration (MBP in *green*) and axonal loss (neurofilament NF-M subtype in *red*) in the spinal cord of the NMO-rat compared to the Control-rat. **d** Increased expression of the NF-H phosphorylated form, a marker of axon injury, detected by Western blot (pNF-H/NF-H ratio; *p* = 0.04). **e** Axon fragmentation and loss in the optic nerve of the NMO-rats compared to the Control-rat detected by NF-M immunostaining. *Scale bars* = 20 μm

**Fig. 7:**
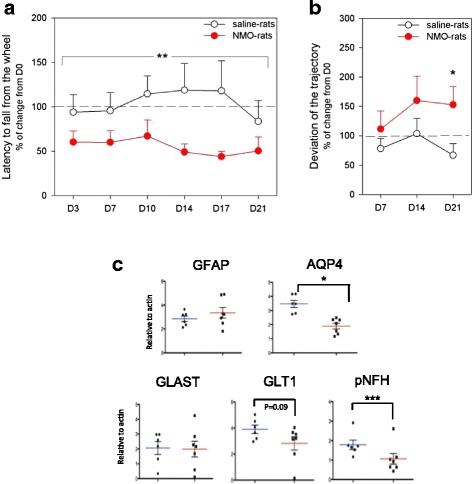
Motor impairment in NMO-rat. Motor skills of the NMO-rats (*red symbols*, *n* = 10, rats infused with IgG^AQP4+^2) as compared to the Saline-rats (*white symbols*, *n* = 10). **a** Rotarod experiment measuring the latency to fall from the rotating rod at D3, D7, D10, D14, and D21 after IgG infusion, expressed as percent of D0 scores (latencies). The NMO-rats showed impairment in this task and fell much sooner from the rod than the Saline-rats. **b** Walking experiment measuring deviation from the linear trajectory initially chosen by the rat at D7, D14, and D21, expressed as percent of D0 shifts (*p < 0.05; **p < 0.01, ANOVA followed by post hoc tests). **c** Western blot analysis of the spinal cord of all rats at D21: altered expression of *AQP4*, *GLT1*, and *pNF-H* in the NMO-rats compared to the Control-rats

We further examined axonal integrity using neurofilament M subunit (NF-M) as axonal marker (Fig. 6). In the WM spinal cord, the number of axons, detected as NF-M-positive spots, appeared less dense in the NMO-rats (*n* = 6) compared to the Control-rats (*n* = 6) (Fig. 6a). Classification (10–20 to 100–140 μm^2^) and quantification (mean by field) of NF-M spots confirmed the loss of axons in the NMO-rats (Fig. 6b, *p* = 0.003), notably the loss of 60–140 μm^2^ axons. In the GM spinal cord of the NMO-rats, axons appeared fragmented and reduced in thickness (Fig. 6a). Axonal loss was associated with myelin disorganization, as shown in the WM by MBP and NF-M co-immunolabeling (Fig. 6c). Western blot analysis of the extensively phosphorylated form of NF-H (pNF-H) in the spinal cord of all rats showed an increased expression of this biomarker of axonal injury [30] in the NMO-rats (Fig. 6d, *p* = 0.04). Axonal alteration was also detected in the optic nerve of the NMO-rats. NF-M-positive axons had reduced thickness and became fragmented (Fig. 6e). Analysis at ultrastructural level (transmission electron microscopy) confirmed the axonal loss in the optic nerve (178.2 ± 15 axons per field (*n* = 11) in the Control-rats versus 118.2 ± 7 in the NMO-rats, *p* = 0.001). Assuming that myelin and axonal alteration may have consequence on motor behavior, we used the rotarod test to investigate motor coordination and a stepping sequence task to evaluate the walking pattern of the NMO-rats (*n* = 10) compared to the controls (*n* = 10) (examination at D3, D7, D10, D14, and D21). At baseline, motor characteristics did not differ in the NMO-rats as compared to the Saline-rats for both rotarod test (67 ± 9 s, *n* = 10 versus 47 ± 11 s, *n* = 11, unpaired *t* test, *p* = 0.1709) and walking deviation experiments (0.4 ± 0.1 cm, *n* = 10 versus 0.5 ± 0.1 cm, *n* = 11, unpaired *t* test, *p* = 0.2694). In the rotarod procedure, the NMO-rats were greatly impaired (Fig. 7a). ANOVAs revealed that the NMO-rats latency to fall was consistently lower than that of the Saline-rats (significant group effect: *F*(1,18) = 6742; *p* = 0.0182). In addition, a post hoc analysis revealed that the NMO-rat latency to fall was significantly lower than their 100 % baseline (performance on D0 prior to surgery—*p* < 0.0001). This was not the case for the Saline-rats (*p* = 0.8244). The NMO-rats performance decreased over days to reach a latency of 50 ± 16 s on D21. The rats were also tested in a non-forced complete stepping sequence task (Fig. 7b). A significant difference was observed at D21 between the NMO-rats and the Saline-rats, with the NMO-rats showing a greater impairment in their walking pattern (deviation from line trajectory) as compared to the Saline-rats (*F*(1,18) = 5.962; *p* = 0.0252). In parallel, alteration of AQP4, GLT1, and pNF-H expression was detected by Western blot in the spinal cord of the NMO-rats (D21) compared to the Saline-rats (Fig. 7c).

### Rat NMO-like lesions are dependent of IgG^AQP4+^

**Fig. 8:**
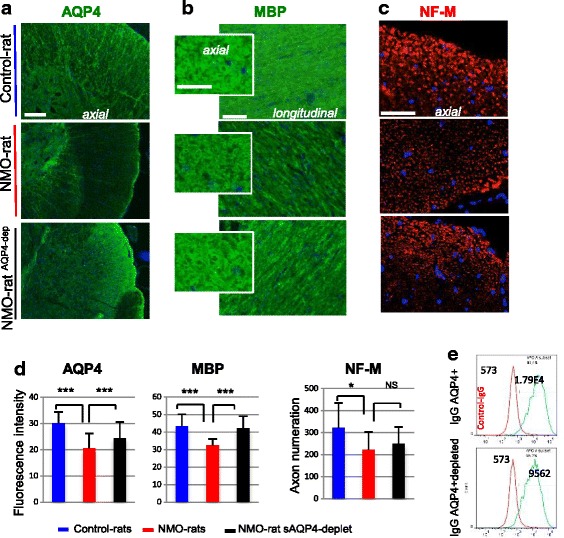
Lesions in the NMO-rat spinal cord depends on the presence of AQP4-Ab. **a**–**c** *AQP4* expression and myelin (*MBP*) and axon (*NF-M*) integrity in the spinal cord (IHC on axial and longitudinal slices, *scale bars* = 20 μm) of the NMO-rat, Control-rat, and rat perfused with purified IgG depleted in IgG^AQP4+^: NMO-rat^AQP4+dep^ (D7). **d** Quantification of fluorescence intensity and axon numeration showed that AQP4-Ab depletion reduced the lesion extend in the spinal cord analyzed for AQP4 (***p < 0.001) and MBP (***p < 0.001). **e** Depletion reduced the amount of AQP4-Ab in IgG^AQP4+dep^ by 40 %, as shown by flow cytometry analysis on HEK-AQP4 cells

To ascertain that the lesions observed in the NMO-rats were directly due to the presence of AQP4-IgG, we infused three rats with IgG^AQP4+^ previously depleted in AQP4-IgG (IgG^AQP4+dep^ adsorption on HEK-AQP4 cells—Fig. 8e). They were compared to rat infused with the non-depleted IgG^AQP4+^ (NMO-rats, *n* = 3). AQP4 expression and myelin and axonal integrity were examined at D7 in the spinal cord using the methods described above. As expected, AQP4 expression decreased in the NMO-rats whereas AQP4-IgG depletion partially prevented such a decrease, as shown by immunodetection (Fig. 8a) and signal quantification (Fig. 8d, NMO-rats^AQP4+dep^ versus NMO-rats, *p* = 0.0001) In parallel, IgG^AQP4+dep^ induced less myelin disorganization and MBP decrease (Fig. 8b, d) than IgG^AQP4+^ (*p* = 0.001) but has less on effect on axonal loss (Fig. 8c, d). All these observations confirmed the direct role of AQP4-IgG in the lesions observed in our study model.

Assuming that IgG infusion in the CNS could induce immune cell infiltration in the rat CNS, we looked for the presence of CD8+, CD45RA+, and CD45+ cells and MPO+ (neutrophil) in the brain, spinal cord, and optic nerve of the NMO-rats (*n* = 4) and the Control-rats (*n* = 4) (D7) by using IHC. The immune cells were detected at the same low level in the NMO-rats and the Control-rats (Additional file 3: Figure S3, CD45+ cells as example). This indicated that perfusion of IgG induced only a mild inflammation. Accordingly, microglia did not display a detectable hallmark of activation or proliferation, as shown by examination of Iba1-positive cells (activated microglia) in the spinal cord and optic nerve (not shown).

In our study model, we were not able, using IHC, to detect perivascular complement deposition in any CNS structure, including the spinal cord and optic nerve. To check the possible role of innate complement activation in lesion formation, we treated rats with cobra venom prior to IgG brain infusion to abolish endogenous complement activation [31]. NMO-like lesions occurred at the same level in the treated or not treated NMO-rats (not shown), demonstrating that complement does not enhance injury in our model.

Globally, NMO-like lesion formation was mainly dependent on the presence of AQP4-IgG but less, if not, on other inflammatory factors.

## Discussion

We herein propose a model, based on prolonged CSF infusion of purified IgG from the serum of AQP4-IgG positive patients, that challenges the intrinsic effect of the autoantibody in NMO physiopathology and ascertain its clinical impact. We show that a prolonged CSF infusion of IgG^AQP4+^ led to the diffusion of antibody in the CNS, including the optic nerve and spinal cord*.* We found that infused IgG^AQP4+^ has the ability to induce astrocyte functional and morphological modification in these structures. Astrocytopathy was associated to myelin modification, to loss of oligodendrocyte and axon, and to clinical motor impairment that could be partly driven by glutamate homeostasis disruption. Our results support that astrocytopathy, myelin and axonal damage reported in NMO, is, to some extent, driven by intrathecal AQP4-IgG itself and in the absence of major complement activation and immune cell infiltration and confirm the role of CSF-AQP4-IgG.

It is now clear that AQP4-IgG are not only a surrogate biomarker but play an important role in NMO pathophysiology. In animal models, intraperitoneal injection of AQP4-IgG in the context of T cell-mediated brain inflammation or intracerebral co-injection of AQP4-IgG and complement induced NMO-like lesions [16–18]. In addition, incubation of the spinal cord slice cultures or primary astrocytes with AQP4-IgG required complement or natural killer cells to demonstrate robust specific cell destruction [19, 20]. These in vitro and animal experiments clearly demonstrated that AQP4-IgG induce astrocytic loss through complement activation, T cell activation, and immune cell cytotoxicity. We reported here that AQP4-IgG per se can also induce astrocytopathy and downstream NMO-like tissue lesions, independent of such neuro-inflammatory processes. We thus showed that histological, biochemical, and clinical changes can be mediated by infused IgG^AQP4+^. Immunoglobulin effect per se was demonstrated since our CNS infusion paradigm did not induce major immune cell infiltration nor microglial cells activation, complement deposit was absent in tissue lesion and complement inhibition by cobra venom did not decrease astrocytopathy. Lack of major perivascular complement deposit in our study model is supported by recent pathology studies reporting heterogeneous type of tissue lesions in NMO [15, 32]. AQP4 antigen specificity was demonstrated by the absence of the effect of the control IgG and the dramatic decrease of the effect in AQP4-IgG-depleted IgG. Our study thus validates a concept that has been already suggested in NMO by several in vitro studies [12–14, 24] and in animal models based on passive transfer of AQP4-IgG in the periphery [33] or iterative injection of AQP4-IgG directly into the spinal cord [21, 34]. There are accumulative evidences for such intrinsic effect of autoantibody against surface antigens in other antibody-mediated CNS disorders. This has been described recently in an animal model of anti-NMDA-receptor-antibody encephalitis based upon a common paradigm of CNS infusion of purified IgG from CSF patients. Planaguma et al. reported a specific targeting of the hippocampus by infused IgG, leading to a downregulation of NMDA-receptor expression and several clinical changes mimicking human disease, in the absence of additional neuro-inflammatory processes [35]. By contrast to this emerging field of autoimmune synaptopathies, characterized by autoantibody permanently present in the CSF, AQP4-IgG is mainly present in the blood [36, 37]. However, the presence of AQP4-IgG in the blood is not sufficient to induce NMO lesion. Indeed AQP4-IgG can be present for years in the periphery without any symptoms [38], and the binding to target antigen in the periphery rarely causes damage [39, 40]. By contrast, AQP4-IgG- and AQP4-producing cells have been identified in the CSF of NMO patients, especially during exacerbation [16, 41]. In established NMO lesions, CNS tissues contain IgG-producing plasma cell. Overall, if the exact role of intrathecally versus peripherally produced AQP4-IgG is not yet clearly established, there is increasing evidence for intrathecal production of AQP4-IgG [42]. For all these reasons, we proposed a NMO model that mimics the presence of AQP4-IgG in the CNS fluid.

Intrathecal IgG^AQP4+^ in rat induces an astrocytopathy that was characterized by a functional alteration rather than an obvious astrocytic death. AQP4 loss, detected in the spinal cord and optic nerve of the NMO-rats, was more intense that demyelination, corroborating the observation that astrocytic alteration precede myelin injury in NMO [6]. We propose a coordinated alteration of AQP4 and membrane-associated astrocytic proteins as key elements of NMO pathogenesis. In particular, we suspect glutamate as a candidate for alterations in NMO because glutamate transporters GLT1/EAAT2 and GLAST/EAAT1 coexist extensively with AQP4 at astrocyte membrane, and a functional link between AQP4 and GLT1 has been reported [43]; AQP4 and GLT1 are downregulated in CNS lesions of NMO patients [13]; and finally, contact with AQP4-IgG leads to GLT1 endocytosis [8, 13] and maintains its sequestration into the cytoplasmic compartment (our data herein). Alteration of glutamate transporters activity could thus result in defective glutamate clearance, an important function preventing glutamate-induced toxicity. We previously shown that chronic contact of the antibody with cultured astrocytes altered glutamate homeostasis (glutamine synthase activity decreased, glutamate release increased) and subsequently damaged oligodendrocytes [24]. In the NMO-rat, we observed a defective glutamate uptake in the rats chronically infused with AQP4-IgG. This metabolic defect was associated with oligodendrocyte loss. A link between altered glutamate homeostasis, myelin defect, and motor deficit may therefore exist in NMO. Glutamate excitotoxicity, secondary to AQP4-IgG-induced alteration in astrocytes, is plausible because glutamate receptors family are present in oligodendrocytes and distributed in myelinated fibers and because astrocytes have the ability to release glutamate and subsequently activate NMDA-receptor, notably in spinal cord, inhibiting or potentiating synaptic activity [44–46]. Astrocytes release glutamate through distinct molecular mechanisms, including vesicular exocytosis and functional hemichannels [47, 48]. In fact, connexin hemichannels, which directly connect the cell cytoplasm to the extracellular space, act as release site for transmitters like glutamate and play a pivotal role in neuron-glial cell crosstalk [49]. In particular, in myelin sheath, connexin-mediated glia networking participates in gliotransmission and impacts myelination and remyelination [50]. Interestingly, a correlation of connexin expression with myelin loss and astrogliosis has been recently reported in NMO lesions [51].

## Conclusions

Our study demonstrates that AQP4-IgG infused in the CSF is, per se, pathogenic in NMO. Our experiments show that some NMO lesions are initiated by AQP4-IgG binding to its target, in the absence of further complement activation, immune cell infiltration, and astrocytic death. The demyelination and axonal loss can be secondary to astrocytopathy, partly driven by glutamate excitotoxicity. We therefore suggest that treatment protecting astrocyte, including dimethyl fumarate [52] or laquinimod [53], could be of major interest in NMO. Our rat NMO model should also be useful for evaluating novel treatments targeting pathogenic autoantibodies, as intravenous IgG, and enzyme targeting and specifically inactivating human IgG antibodies.

## Ethics approval and consent to participate

Animal experiments were carried out in strict accordance with the European Directive 2010/63/UE and follows the ARRIVE guidelines on the protection of animals used for scientific purposes. Adult male OFA rats at 280 g (Janvier; Le Genest Saint Isle, France) were acclimated to the new environment for 7 days prior to the start of the experiment. The local Lyon 1 University Animal Care Committee has approved all the experimental procedures on the animals (BH2012-80 project).

### Consent for publication

Not applicable.

### Availability of data and materials

Relevant raw data are freely available.

## Additional files

Additional file 1: Figure S1. Characterization of IgG^AQP4+^ used for rat brain infusion. **A** IgG^AQP4+^ binding (green) on spinal cord and optic nerve from naïve rat (longitudinal sections, immunofluorescence, IgG^AQP4+^ 2 as example): co-localization with GFAP (red) and AQP4 (red) on astrocyte processes. Scale bar = 20 μm. **B**, **C** Reduction of AQP4 in total cell lysate (B) and membrane preparation (C) of cultured astrocytes following 24 h contact with IgG^AQP4+^; IgG^Control^ had no similar effect (Western blot, actin as control for protein deposition). Additional file 2: Figure S2. Stepping sequence analysis. **A** Weight curves of NMO-rats and Control-rats during motor behavior analyses. **B** Deviation from the linear trajectory initially chosen by the rat once it entered the corridor was computed and scored, using the left forepaw prints. Additional file 3: Figure S3. Low immune cell infiltration in NMO-rat. CD45+ lymphocytes infiltrated in the brain, optic nerve, and spinal cord of NMO-rat and Control-rat at low level (D7). Scale bar = 20 μm.

## Abbreviations

AQP4: aquaporin-4
AQP4-IgG: aquaporin-4 autoantibody
CNS: central nervous system
CSF: cerebrospinal fluid
GFAP: glial fibrillary acidic protein
GLAST: glutamate aspartate transporter
GLT1: glutamate transporter type 1
GM: gray matter
IgG: immunoglobulins
IHC: immunohistochemistry
MBP: myelin basic protein
NF: neurofilament
NMO: Devic’s neuromyelitis optica
PLP: proteolipid protein
TEM: transmission electron microscopy
WM: white matter

## References

[1] Sharma R, Fischer MT, Bauer J, Felts PA, Smith KJ, Misu T, Fujihara K, Bradl M, Lassmann H (2010). Inflammation induced by innate immunity in the central nervous system leads to primary astrocyte dysfunction followed by demyelination. Acta Neuropathol.

[2] Takano R, Misu T, Takahashi T, Sato S, Fujihara K, Itoyama Y (2010). Astrocytic damage is far more severe than demyelination in NMO: a clinical CSF biomarker study. Neurology.

[3] Kuroda H, Fujihara K, Takano R, Takai Y, Takahashi T, Misu T, Nakashima I, Sato S, Itoyama Y, Aoki M (2013). Increase of complement fragment C5a in cerebrospinal fluid during exacerbation of neuromyelitis optica. J Neuroimmunol.

[4] Howe CL, Kaptzan T, Magana SM, Ayers-Ringler JR, LaFrance-Corey RG, Lucchinetti CF (2014). Neuromyelitis optica IgG stimulates an immunological response in rat astrocyte cultures. Glia.

[5] Lucchinetti CF, Guo Y, Popescu BF, Fujihara K, Itoyama Y, Misu T (2014). The pathology of an autoimmune astrocytopathy: lessons learned from neuromyelitis optica. Brain Pathol.

[6] Roemer SF, Parisi JE, Lennon VA, Benarroch EE, Lassmann H, Bruck W, Mandler RN, Weinshenker BG, Pittock SJ, Wingerchuk DM, Lucchinetti CF (2007). Pattern-specific loss of aquaporin-4 immunoreactivity distinguishes neuromyelitis optica from multiple sclerosis. Brain.

[7] Misu T, Fujihara K, Kakita A, Konno H, Nakamura M, Watanabe S, Takahashi T, Nakashima I, Takahashi H, Itoyama Y (2007). Loss of aquaporin 4 in lesions of neuromyelitis optica: distinction from multiple sclerosis. Brain.

[8] Hinson SR, Pittock SJ, Lucchinetti CF, Roemer SF, Fryer JP, Kryzer TJ, Lennon VA (2007). Pathogenic potential of IgG binding to water channel extracellular domain in neuromyelitis optica. Neurology.

[9] Sabater L, Giralt A, Boronat A, Hankiewicz K, Blanco Y, Llufriu S, Alberch J, Graus F, Saiz A (2009). Cytotoxic effect of neuromyelitis optica antibody (NMO-IgG) to astrocytes: an in vitro study. J Neuroimmunol.

[10] Diamond B, Huerta PT, Mina-Osorio P, Kowal C, Volpe BT (2009). Losing your nerves? Maybe it’s the antibodies. Nat Rev Immunol.

[11] Popescu BF, Lucchinetti CF (2012). Pathology of demyelinating diseases. Annu Rev Pathol.

[12] Vincent T, Saikali P, Cayrol R, Roth AD, Bar-Or A, Prat A, Antel JP (2008). Functional consequences of neuromyelitis optica-IgG astrocyte interactions on blood-brain barrier permeability and granulocyte recruitment. J Immunol.

[13] Hinson SR, Roemer SF, Lucchinetti CF, Fryer JP, Kryzer TJ, Chamberlain JL, Howe CL, Pittock SJ, Lennon VA (2008). Aquaporin-4-binding autoantibodies in patients with neuromyelitis optica impair glutamate transport by down-regulating EAAT2. J Exp Med.

[14] Hinson SR, Romero MF, Popescu BF, Lucchinetti CF, Fryer JP, Wolburg H, Fallier-Becker P, Noell S, Lennon VA (2012). Molecular outcomes of neuromyelitis optica (NMO)-IgG binding to aquaporin-4 in astrocytes. Proc Natl Acad Sci U S A.

[15] Misu T, Hoftberger R, Fujihara K, Wimmer I, Takai Y, Nishiyama S, Nakashima I, Konno H, Bradl M, Garzuly F (2013). Presence of six different lesion types suggests diverse mechanisms of tissue injury in neuromyelitis optica. Acta Neuropathol.

[16] Bennett JL, Lam C, Kalluri SR, Saikali P, Bautista K, Dupree C, Glogowska M, Case D, Antel JP, Owens GP (2009). Intrathecal pathogenic anti-aquaporin-4 antibodies in early neuromyelitis optica. Ann Neurol.

[17] Bradl M, Misu T, Takahashi T, Watanabe M, Mader S, Reindl M, Adzemovic M, Bauer J, Berger T, Fujihara K (2009). Neuromyelitis optica: pathogenicity of patient immunoglobulin in vivo. Ann Neurol.

[18] Saadoun S, Waters P, Bell BA, Vincent A, Verkman AS, Papadopoulos MC (2010). Intra-cerebral injection of neuromyelitis optica immunoglobulin G and human complement produces neuromyelitis optica lesions in mice. Brain.

[19] Ratelade J, Verkman AS (2012). Neuromyelitis optica: aquaporin-4 based pathogenesis mechanisms and new therapies. Int J Biochem Cell Biol.

[20] Zhang H, Bennett JL, Verkman AS (2011). Ex vivo spinal cord slice model of neuromyelitis optica reveals novel immunopathogenic mechanisms. Ann Neurol.

[21] Geis C, Ritter C, Ruschil C, Weishaupt A, Grunewald B, Stoll G, Holmoy T, Misu T, Fujihara K, Hemmer B (2015). The intrinsic pathogenic role of autoantibodies to aquaporin 4 mediating spinal cord disease in a rat passive-transfer model. Exp Neurol.

[22] Wingerchuk DM (2007). Diagnosis and treatment of neuromyelitis optica. Neurologist.

[23] Marignier R, Bernard-Valnet R, Giraudon P, Collongues N, Papeix C, Zephir H, Cavillon G, Rogemond V, Casey R, Frangoulis B (2013). Aquaporin-4 antibody-negative neuromyelitis optica: distinct assay sensitivity-dependent entity. Neurology.

[24] Marignier R, Nicolle A, Watrin C, Touret M, Cavagna S, Varrin-Doyer M, Cavillon G, Rogemond V, Confavreux C, Honnorat J, Giraudon P (2010). Oligodendrocytes are damaged by neuromyelitis optica immunoglobulin G via astrocyte injury. Brain.

[25] Varrin-Doyer M, Nicolle A, Marignier R, Cavagna S, Benetollo C, Wattel E, Giraudon P (2012). Human T lymphotropic virus type 1 increases T lymphocyte migration by recruiting the cytoskeleton organizer CRMP2. J Immunol.

[26] Touret M, Parrot S, Denoroy L, Belin MF, Didier-Bazes M (2007). Glutamatergic alterations in the cortex of genetic absence epilepsy rats. BMC Neurosci.

[27] Bert L, Favale D, Jego G, Greve P, Guilloux JP, Guiard BP, Gardier AM, Suaud-Chagny MF, Lestage P (2004). Rapid and precise method to locate microdialysis probe implantation in the rodent brain. J Neurosci Methods.

[28] Nolan MF, Malleret G, Lee KH, Gibbs E, Dudman JT, Santoro B, Yin D, Thompson RF, Siegelbaum SA, Kandel ER, Morozov A (2003). The hyperpolarization-activated HCN1 channel is important for motor learning and neuronal integration by cerebellar Purkinje cells. Cell.

[29] Metz GA, Whishaw IQ (2002). Drug-induced rotation intensity in unilateral dopamine-depleted rats is not correlated with end point or qualitative measures of forelimb or hindlimb motor performance. Neuroscience.

[30] Petzold A, Tozer DJ, Schmierer K (2011). Axonal damage in the making: neurofilament phosphorylation, proton mobility and magnetisation transfer in multiple sclerosis normal appearing white matter. Exp Neurol.

[31] Asavapanumas N, Ratelade J, Papadopoulos MC, Bennett JL, Levin MH, Verkman AS (2014). Experimental mouse model of optic neuritis with inflammatory demyelination produced by passive transfer of neuromyelitis optica-immunoglobulin G. J Neuroinflammation.

[32] Saji E, Arakawa M, Yanagawa K, Toyoshima Y, Yokoseki A, Okamoto K, Otsuki M, Akazawa K, Kakita A, Takahashi H (2013). Cognitive impairment and cortical degeneration in neuromyelitis optica. Ann Neurol.

[33] Chan KH, Zhang R, Kwan JS, Guo VY, Ho PW, Ho JW, Chu AC (2012). Aquaporin-4 autoantibodies cause asymptomatic aquaporin-4 loss and activate astrocytes in mouse. J Neuroimmunol.

[34] Geis C (2014). Effects of pooled human immunoglobulins in an animal model of neuromyelitis optica with chronic application of autoantibodies to aquaporin 4. Clin Exp Immunol.

[35] Planaguma J, Leypoldt F, Mannara F, Gutierrez-Cuesta J, Martin-Garcia E, Aguilar E, Titulaer MJ, Petit-Pedrol M, Jain A, Balice-Gordon R (2015). Human N-methyl D-aspartate receptor antibodies alter memory and behaviour in mice. Brain.

[36] Jarius S, Franciotta D, Paul F, Ruprecht K, Bergamaschi R, Rommer PS, Reuss R, Probst C, Kristoferitsch W, Wandinger KP, Wildemann B (2010). Cerebrospinal fluid antibodies to aquaporin-4 in neuromyelitis optica and related disorders: frequency, origin, and diagnostic relevance. J Neuroinflammation.

[37] Dujmovic I, Mader S, Schanda K, Deisenhammer F, Stojsavljevic N, Kostic J, Berger T, Drulovic J, Reindl M (2011). Temporal dynamics of cerebrospinal fluid anti-aquaporin-4 antibodies in patients with neuromyelitis optica spectrum disorders. J Neuroimmunol.

[38] Nishiyama S, Ito T, Misu T, Takahashi T, Kikuchi A, Suzuki N, Jin K, Aoki M, Fujihara K, Itoyama Y (2009). A case of NMO seropositive for aquaporin-4 antibody more than 10 years before onset. Neurology.

[39] Kitic M, Hochmeister S, Wimmer I, Bauer J, Misu T, Mader S, Reindl M, Fujihara K, Lassmann H, Bradl M (2013). Intrastriatal injection of interleukin-1 beta triggers the formation of neuromyelitis optica-like lesions in NMO-IgG seropositive rats. Acta Neuropathol Commun.

[40] Pohl M, Kawakami N, Kitic M, Bauer J, Martins R, Fischer MT, Machado-Santos J, Mader S, Ellwart JW, Misu T (2013). T cell-activation in neuromyelitis optica lesions plays a role in their formation. Acta Neuropathol Commun.

[41] Chihara N, Aranami T, Sato W, Miyazaki Y, Miyake S, Okamoto T, Ogawa M, Toda T, Yamamura T (2011). Interleukin 6 signaling promotes anti-aquaporin 4 autoantibody production from plasmablasts in neuromyelitis optica. Proc Natl Acad Sci U S A.

[42] Zekeridou A, Lennon VA (2015). Aquaporin-4 autoimmunity. Neurol Neuroimmunol Neuroinflamm.

[43] Li YK, Wang F, Wang W, Luo Y, Wu PF, Xiao JL, Hu ZL, Jin Y, Hu G, Chen JG (2012). Aquaporin-4 deficiency impairs synaptic plasticity and associative fear memory in the lateral amygdala: involvement of downregulation of glutamate transporter-1 expression. Neuropsychopharmacology.

[44] Talantova M, Sanz-Blasco S, Zhang X, Xia P, Akhtar MW, Okamoto S, Dziewczapolski G, Nakamura T, Cao G, Pratt AE (2013). Abeta induces astrocytic glutamate release, extrasynaptic NMDA receptor activation, and synaptic loss. Proc Natl Acad Sci U S A.

[45] De Pitta M, Volman V, Berry H, Ben-Jacob E (2011). A tale of two stories: astrocyte regulation of synaptic depression and facilitation. PLoS Comput Biol.

[46] Ficker C, Rozmer K, Kato E, Ando RD, Schumann L, Krugel U, Franke H, Sperlagh B, Riedel T, Illes P (2014). Astrocyte-neuron interaction in the substantia gelatinosa of the spinal cord dorsal horn via P2X7 receptor-mediated release of glutamate and reactive oxygen species. Glia.

[47] Parpura V, Verkhratsky A (2012). Astrocytes revisited: concise historic outlook on glutamate homeostasis and signaling. Croat Med J.

[48] Orellana JA, Stehberg J (2014). Hemichannels: new roles in astroglial function. Front Physiol.

[49] Ye ZC, Wyeth MS, Baltan-Tekkok S, Ransom BR (2003). Functional hemichannels in astrocytes: a novel mechanism of glutamate release. J Neurosci.

[50] Li T, Giaume C, Xiao L (2014). Connexins-mediated glia networking impacts myelination and remyelination in the central nervous system. Mol Neurobiol.

[51] Masaki K, Suzuki SO, Matsushita T, Matsuoka T, Imamura S, Yamasaki R, Suzuki M, Suenaga T, Iwaki T, Kira J (2013). Connexin 43 astrocytopathy linked to rapidly progressive multiple sclerosis and neuromyelitis optica. PLoS One.

[52] Linker RA, Lee DH, Ryan S, van Dam AM, Conrad R, Bista P, Zeng W, Hronowsky X, Buko A, Chollate S (2011). Fumaric acid esters exert neuroprotective effects in neuroinflammation via activation of the Nrf2 antioxidant pathway. Brain.

[53] Bruck W, Zamvil SS (2012). Laquinimod, a once-daily oral drug in development for the treatment of relapsing-remitting multiple sclerosis. Expert Rev Clin Pharmacol.

