# Supplementary figures and images for: Neuromyelitis optica study model based on chronic infusion of autoantibodies in rat cerebrospinal fluid
Source: J Neuroinflammation. 2016 May 18;13:111. doi: 10.1186/s12974-016-0577-8 (PMC4872335; doi:10.1186/s12974-016-0577-8)

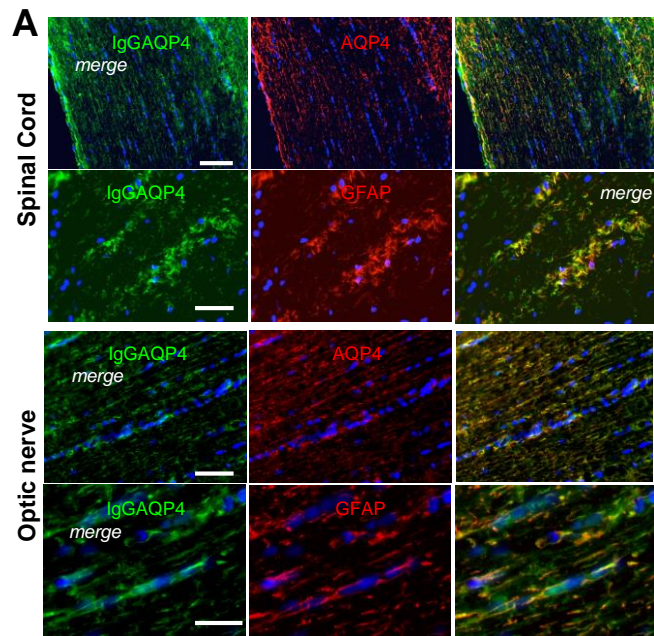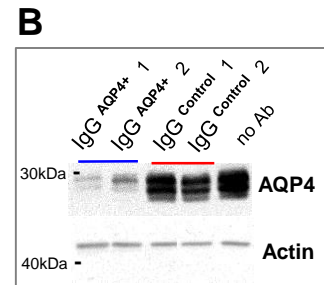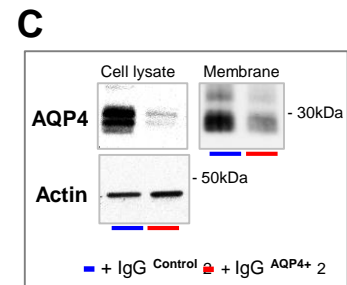

Supplement: Additional file 1: Figure S1. — Characterization of IgGAQP4+ used for rat brain infusion. A IgGAQP4+ binding (green) on spinal cord and optic nerve from naïve rat (longitudinal sections, immunofluorescence, IgGAQP4+ 2 as example): co-localization with GFAP (red) and AQP4 (red) on astrocyte processes. Scale bar = 20 μm. B, C Reduction of AQP4 in total cell lysate (B) and membrane preparation (C) of cultured astrocytes following 24 h contact with IgGAQP4+; IgGControl had no similar effect (Western blot, actin as control for protein deposition). [file 12974_2016_577_MOESM1_ESM.pdf]

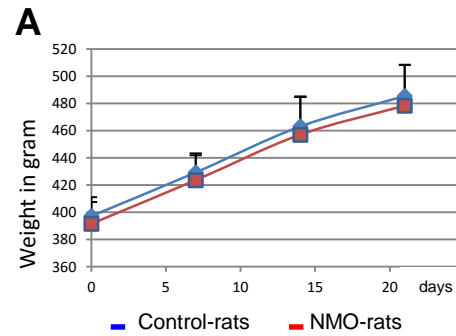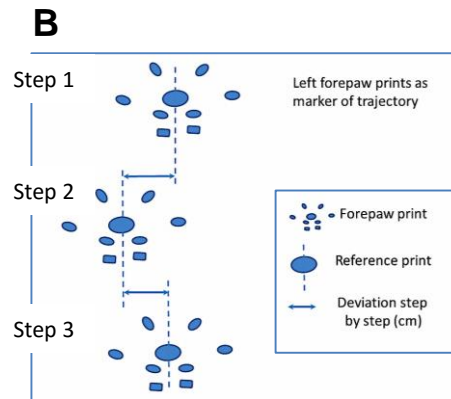

Supplement: Additional file 2: Figure S2. — Stepping sequence analysis. A Weight curves of NMO-rats and Control-rats during motor behavior analyses. B Deviation from the linear trajectory initially chosen by the rat once it entered the corridor was computed and scored, using the left forepaw prints. [file 12974_2016_577_MOESM2_ESM.pdf]

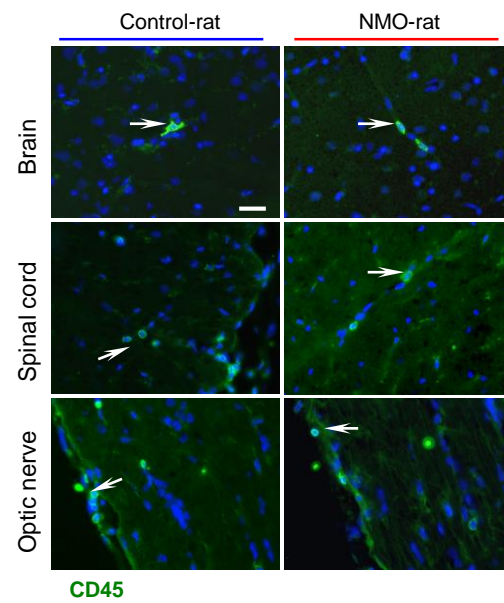

Supplement: Additional file 3: Figure S3. — Low immune cell infiltration in NMO-rat. CD45+ lymphocytes infiltrated in the brain, optic nerve, and spinal cord of NMO-rat and Control-rat at low level (D7). Scale bar = 20 μm. [file 12974_2016_577_MOESM3_ESM.pdf]
